# Crystal structures of 1-hy­droxy-4-prop­yloxy-9,10-anthra­quinone and its acetyl derivative

**DOI:** 10.1107/S2056989017015973

**Published:** 2017-11-10

**Authors:** Hidemi Nakagawa, Chitoshi Kitamura

**Affiliations:** a Department of Materials Science, School of Engineering, The University of Shiga Prefecture, 2500 Hassaka-cho, Hikone, Shiga 522-8533, Japan

**Keywords:** crystal structure, anthra­quinone, hydrogen bonding, π–π stacking, C—H ⋯O inter­actions

## Abstract

1-Hy­droxy-4-prop­yloxy-9,10-anthra­quinone, C_17_H_14_O_4_, (I), and its acetyl derivative, 4-acet­yloxy-4-prop­yloxy-9,10-anthra­quinone, C_19_H_16_O_5_, (II), were synthesized from the commercially available dye quinizarin. In both compounds, the anthra­quinone frameworks are close to planarity. There is a large difference in the conformation of the prop­yloxy group; the mol­ecule of (I) adopts a *gauche* conformation [O—C—C—C = −64.4 (2)°], although the mol­ecule of (II) takes a *trans*-planar conformation (zigzag) [O—C—C—C = 176.1 (3)°]. In the mol­ecule of (I), there is an intra­molecular O—H⋯O hydrogen bond. In both crystals, the mol­ecules are linked by C—H ⋯O hydrogen bonds. A difference in the mol­ecular arrangements of (I) and (II) is found along the stacking directions.

## Abstract 2

The title compounds were synthesized from the commercially available dye quinizarin. In both compounds, the anthra­quinone frameworks are close to planarity but there is a large difference in the conformation of the prop­yloxy group.

## Chemical context   

9,10-Anthra­quinone and its derivatives are important mol­ecules as dyes and pigments. As a part of a project on the study of the substitution effects of the anthra­quinone ring on optical properties in solution as well as in the solid state, we have been synthesizing new anthra­quinone derivatives. Recently, we found that the recrystallization of 1,4-diprop­yloxy-9,10-antha­quinone from hexane solution afforded two polymorphs, red prisms and yellow needles, whose crystal structures were different from each other (Kitamura *et al.*, 2015*b* ▸). Then we became inter­ested in the effect of the asymmetric substitution pattern of 9,10-anthra­quinone because 1,4-diprop­yloxy-9,10-anthra­quinone is a symmetric mol­ecule along the direction of the mol­ecular short axis. We thought that mono-alk­oxy­lation from quinizarin (1,4-dihy­droxy-9,10-anthra­quinone) should be effective to gain asymmetric 9,10-anthra­quinones along the mol­ecular short axis. We report herein the synthesis and crystal structures of 1-hy­droxy-4-prop­yloxy-9,10-anthra­quinone (I) and its acetyl derivative, 1-acet­yloxy-4-prop­yloxy-9,10-anthra­quinone (II).
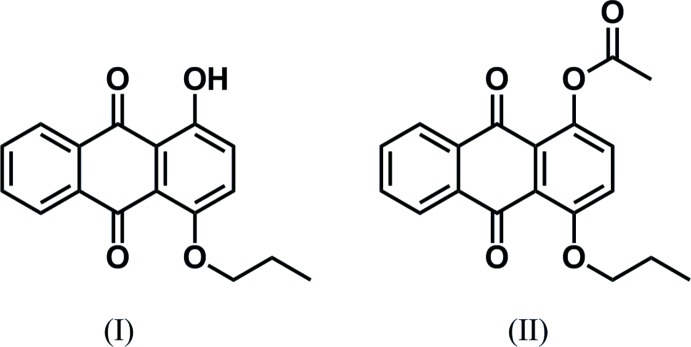


## Structural commentary   

**Figure 1:**
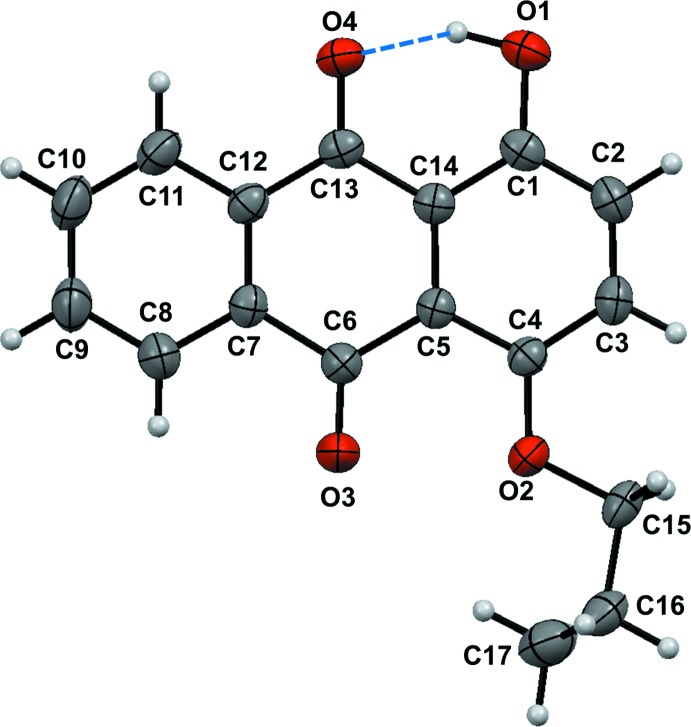
Mol­ecular structure of compound (I), showing the atom labelling and 50% probability displacement ellipsoids. The intra­molecular hydrogen bond is indicated by a dashed line.

The mol­ecular structures of the title compounds, (I) and (II), are illustrated in Figs. 1 ▸ and 2 ▸, respectively. In both mol­ecules, the anthra­quinone frameworks are nearly planar. However, there is a large difference in the conformation of the prop­yloxy group; in compound (I), the the prop­yloxy moiety adopts a *gauche* conformation [O2—C15—C16—C17 torsion angle = 64.4 (2)°], and in compound (II), it has a *trans*-planar (zigzag) conformation [O2—C17—C18—C19 = 176.1 (3)°]. In (I), there is an intra­molecular O—H⋯O hydrogen bond forming an *S*(6) ring motif (Fig. 1 ▸ and Table 1 ▸). In compound (II), the acetyl group plane (O1/O5/C15/C16) is inclined to the anthra­quinone ring system by 71.87 (12)°.

## Supra­molecular features   

**Figure 3:**
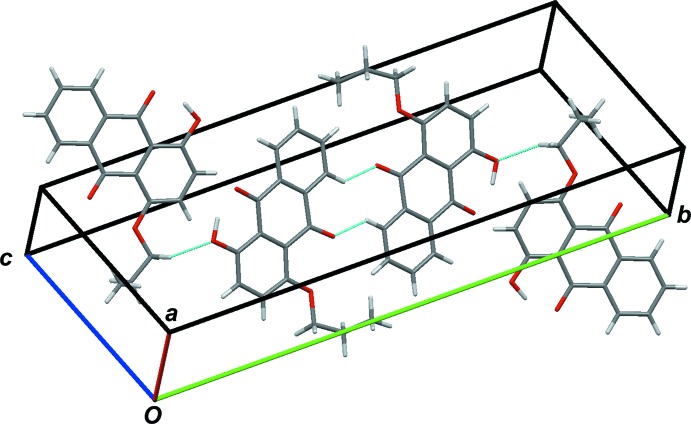
Packing of the unit cell of (I), showing short C—H⋯O contacts as blue lines.

**Figure 4:**
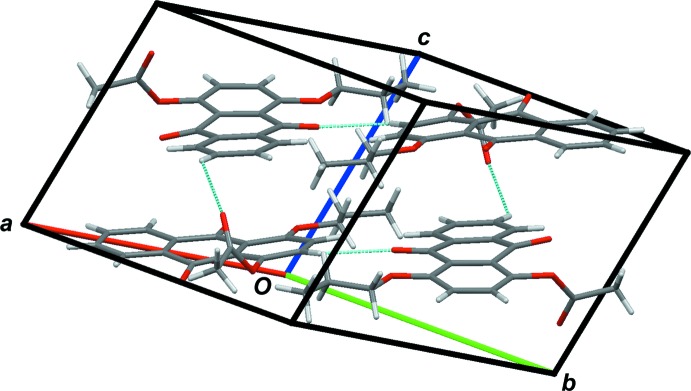
Packing of the unit cell of (II), showing short C—H⋯O contacts as blue lines.

**Figure 5:**
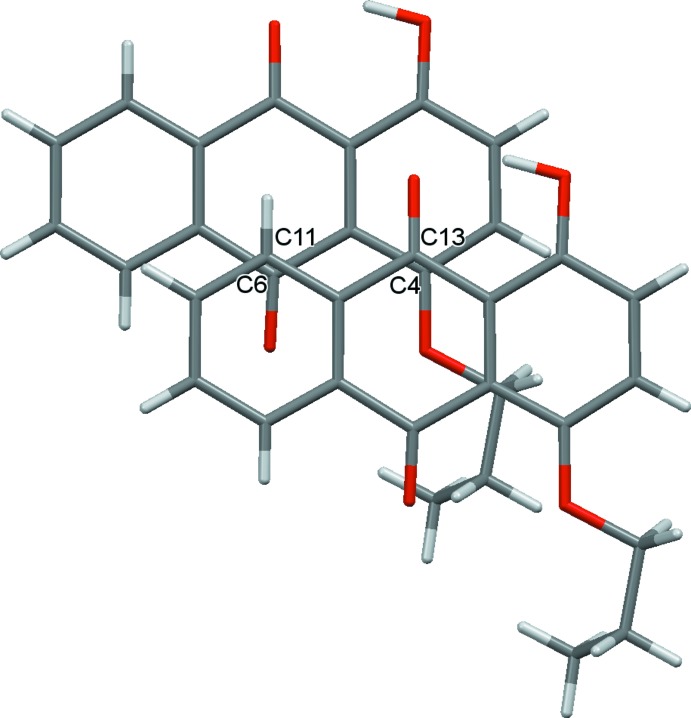
Top view of two neighboring mol­ecules of compound (I) along the stacking direction.

**Figure 7:**
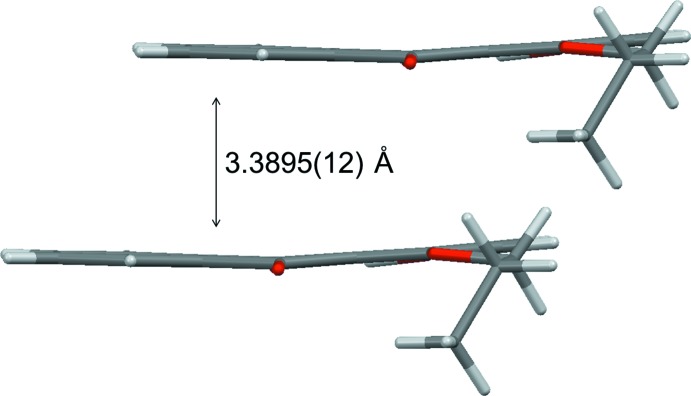
Side view of two neighboring mol­ecules of compound (I).

The crystal packing structures of the title compounds, (I) and (II), are shown in Figs. 3 ▸ and 4 ▸, respectively. In both crystals, mol­ecules are linked by inter­molecular C—H⋯O hydrogen bonds. For compound (I), C—H⋯O hydrogen bonds along the lateral direction of the mol­ecules are found (Fig. 3 ▸ and Table 1 ▸): C8—H8⋯O3^i^, C15—H15*A*⋯O1^ii^ [symmetry codes: (i) −*x* + 1, −*y* + 1, −*z* + 1; (ii) *x* − 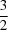, −*y* + 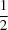, *z* − 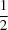]. In contrast, in compound (II) C—H⋯O inter­actions are formed along all directions (Fig. 4 ▸ and Table 2 ▸): C2—H2⋯O3^iii^, C10—H10⋯O5^iv^ [symmetry codes: (iii) −*x* + 1, *y* − 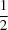, −*z* + 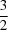; (iv) *x*, −*y* + 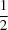, *z* − 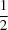] . To understand the solid-state optical properties of dyes, revealing the characteristics of the stacking patterns of neighboring mol­ecules is important. In both crystals, the anthra­quinone ring systems are arranged nearly parallel, although there is a difference in the mol­ecular arrangement of two neighboring mol­ecules along the stacking directions (Figs. 5 ▸–8 ▸ ▸ ▸). As shown in Figs. 5 ▸ and 6 ▸, a small π overlap of the anthra­quinone ring systems is observed for compound (II), on the other hand, compound (I) scarcely shows any π overlap. Regarding the overlap of the anthra­quinone ring systems, in compound (I) there is a translational slip, while in compound (II) there is a rotational slip. The shortest distances for overlapping non-bonded atoms in the anthra­quinone frameworks are 3.297 (2) Å (C11⋯C6^v^) and 3.558 (2) Å (C13⋯C4^v^) in compound (I), and 3.363 (4) Å (C8⋯C4^iv^), 3.423 (4) Å (C11⋯C6^iv^) and 3.523 (4) Å (C10⋯C14^iv^) in compound (II) [symmetry code: (v) *x* + 1, *y*, *z*]. As shown in Figs. 7 ▸ and 8 ▸, the inter­planar distances between the anthra­quinone planes [3.3895 (12) Å for compound (I) and 3.396 (3) Å for compound (II)] are almost identical. The degree of overlap and the inter­planar distance between two chromophores are considered to be the two factors essential for evaluating inter­molecular inter­actions. Therefore compound (II) would have stronger inter­molecular inter­actions than compound (I).

## Database survey   

A literature search found no reports of crystal structures of 1-hy­droxy-4-prop­yloxy-9,10-anthra­quinone (I) and 1-acet­yloxy-4-prop­yloxy-9,10-anthra­quinone (II). Other hy­droxy- or alk­oxy-substituted anthra­quinone compounds have been reported: 4-(3-bromo­prop­yloxy)-1-hy­droxy-9,10-anthra­quin­one (Ohira *et al.*, 2016 ▸), 1,4-diprop­yloxy-9,10-anthra­quinone (Kitamura *et al.*, 2015*b* ▸), 1,4-dihy­droxy-2,3-di­nitro-9,10-anthra­quinone (Furukawa *et al.*, 2016 ▸), 1,4-dieth­oxy-9,10-anthra­quinone (Kitamura *et al.*, 2015*a* ▸), 2-bromo-1,4-dihy­droxy-9,10-anthra­quinone (Furukawa *et al.*, 2014 ▸), 2,6-dimeth­oxy-9,10-anthra­quinone (Ohta *et al.*, 2012*a* ▸), 2,6-diprop­yloxy-9,10-anthra­quinone (Ohta *et al.*, 2012*b* ▸), 2,3,6,7-tetra­prop­yloxy-9,10-anthra­quinone (Ohta *et al.*, 2012*b* ▸).

## Synthesis and crystallization   

**Figure 9:**
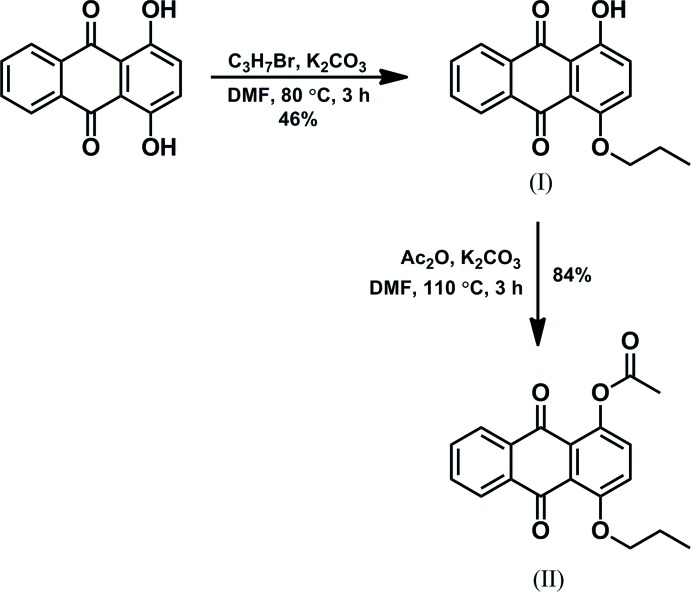
Reaction scheme for the synthesis of compounds (I) and (II).

The title compounds, (I) and (II), were synthesized starting from quinizarin (1,4-dihy­droxy-9,10-anthra­quinone), as shown in Fig. 9 ▸

**Compound (I):** A mixture of quinizarin (289 mg, 1.20 mmol), 1-bromo­propane (675 mg, 5.49 mmol), K_2_CO_3_ (185 mg, 1.34 mmol) in DMF (5 mL) was stirred at 353 K for 3 h under N_2_. After cooling to room temperature, water (60 mL) was added to the reaction mixture. The brown solid that precipitated was filtered off. The resulting solid was solubilized with CH_2_Cl_2._ The organic layer was washed with 1 *M* NaOH to remove the unreacted quinizarin, then washed sequentially with brine, dried over Na_2_SO_4_, and evaporated under reduced pressure. The residual brown solid was purified by chromatography on silica gel with an eluent of CH_2_Cl_2_. The title compound (I) was obtained as an orange solid (132 mg, 46%). m.p. 387.5–389 K. ^1^H NMR (400 MHz, CDCl_3_): *δ* 1.14 (*t*, *J* = 7.4 Hz, 3H, CH_3_), 1.91–2.00 (*m*, 2H, CH_2_), 4.11 (*t*, *J* = 6.6 Hz, 2H, CH_2_), 7.28–7.32 (*m*, 1H, ArH), 7.39–7.41 (*m*, 1H, ArH), 7.73–7.82 (*m*, 2H, ArH), 8.27–8.31 (*m*, 2H, ArH), 13.03 (*s*, 1H, OH). Crystals suitable for X-ray diffraction were grown by slow evaporation of an AcOEt–hexane (*>v*:*v* = 1:10) solution.

**Compound (II):** A mixture of compound (I) (132 mg, 0.47 mmol), K_2_CO_3_ (137 mg, 0.99 mmol) in acetic anhydride (5 mL) was stirred at 383 K for 3 h under air. After cooling to room temperature, water (50 mL) was added into the resulting mixture, then the mixture was stirred for 20 min at room temperature. The mixture was extracted with CH_2_Cl_2_. The organic layer was washed with 10% NaHCO_3_ solution and then brine, and dried over Na_2_SO_4_, and evaporated under reduced pressure. The residual yellow solid was purified by recrystallization from a hexa­ne–toluene (*>v*:*v* = 3:1) solution to provide title compound (II) as a yellow solid (128 mg, 84%). m.p. 401–403 K. ^1^H NMR (400 MHz, CDCl_3_): *δ* 1.146 (*t*, *J* = 7.3 Hz, 3H, CH_3_), 1.93–2.02 (*m*, 2H, CH_2_), 2.48 (*s*, 3H, CH_3_), 4.13 (*t*, *J* = 6.4 Hz, 2H, CH_2_), 7.32–7.36 (*m*, 2H, ArH), 7.68–7.76 (*m*, 2H, ArH), 8.12–8.22 (*m*, 2H, ArH). Crystals suitable for X-ray diffraction were grown by slow evaporation of a hexane-toluene (*>v*:*v* = 18:1) solution.

## Refinement   

Crystal data, data collection and structure refinement details are summarized in Table 3 ▸. The hydroxyl H atom, H1 of compound (I), was refined isotropically. All other H atoms were positioned geometrically and treated as riding atoms: C—H = 0.95–0.99 Å with *U*_iso_(H) = 1.5*U*_eq_(C) for CH_3_ and 1.2*U*_eq_(C) for CH_2_ and aromatic C—H.

## Supplementary Material

Crystal structure: contains datablock(s) global, I, II. DOI: 10.1107/S2056989017015973/nr2068sup1.cif

Structure factors: contains datablock(s) I. DOI: 10.1107/S2056989017015973/nr2068Isup2.hkl

Structure factors: contains datablock(s) II. DOI: 10.1107/S2056989017015973/nr2068IIsup3.hkl

Supporting information file. DOI: 10.1107/S2056989017015973/nr2068Isup4.cml

Supporting information file. DOI: 10.1107/S2056989017015973/nr2068IIsup5.cml

CCDC references: 1583677, 1583676

Additional supporting information:  crystallographic information; 3D view; checkCIF report

## Figures and Tables

**Figure 2:**
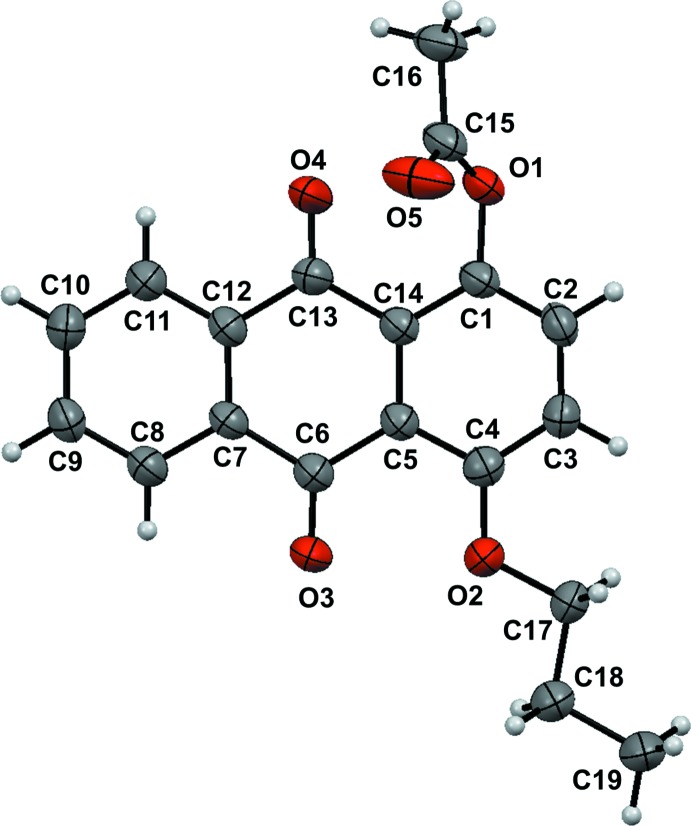
Mol­ecular structure of compound (II), showing the atom labelling and 50% probability displacement ellipsoids.

**Figure 6:**
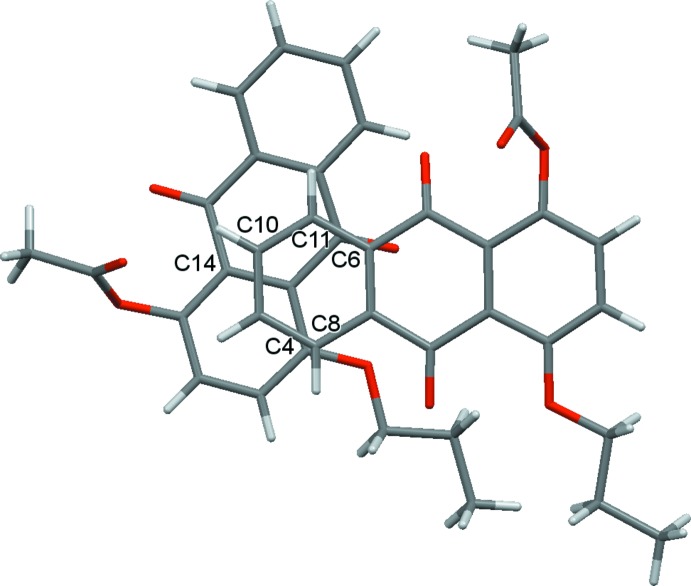
Top view of two neighboring mol­ecules of compound (II) along the stacking direction.

**Figure 8:**
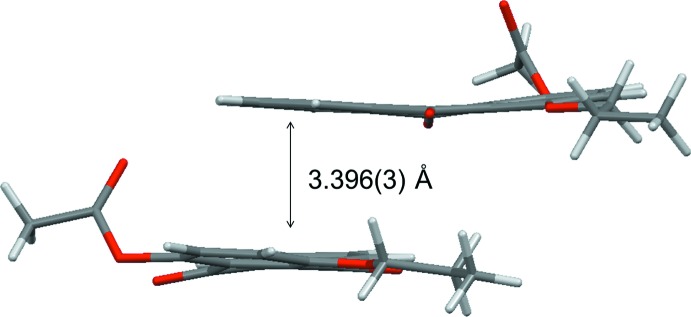
Side view of two neighboring mol­ecules of compound (II).

**Table 1:** Hydrogen-bond geometry (Å, °) for (I)

*D*—H⋯*A*	*D*—H	H⋯*A*	*D*⋯*A*	*D*—H⋯*A*
O1—H1⋯O4	0.98 (3)	1.61 (3)	2.525 (2)	153 (2)
C8—H8⋯O3^i^	0.95	2.51	3.270 (2)	137
C15—H15*A*⋯O1^ii^	0.99	2.88	3.359 (2)	111

Symmetry codes: (i) 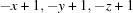; (ii) 
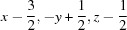
.

**Table 2:** Hydrogen-bond geometry (Å, °) for (II)

*D*—H⋯*A*	*D*—H	H⋯*A*	*D*⋯*A*	*D*—H⋯*A*
C2—H2⋯O3^i^	0.95	2.6	3.384 (4)	140
C10—H10⋯O5^ii^	0.95	2.57	3.180 (4)	123

Symmetry codes: (i) 
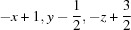
; (ii) 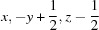.

**Table 3:** Experimental details

	(I)	(II)
Crystal data		
Chemical formula	C_17_H_14_O_4_	C_19_H_16_O_5_
*M*_r_	282.28	324.32
Crystal system, space group	Monoclinic, *P*2_1_/*n*	Monoclinic, *P*2_1_/*c*
Temperature (K)	200	200
*a*, *b*, *c* (Å)	4.7354 (3), 25.9882 (17), 11.0671 (9)	11.7730 (12), 15.514 (2), 8.9609 (10)
β (°)	102.268 (7)	111.153 (8)
*V* (Å^3^)	1330.87 (17)	1526.4 (3)
*Z*	4	4
Radiation type	Mo *K*α	Mo *K*α
μ (mm^−1^)	0.1	0.10
Crystal size (mm)	0.5 × 0.13 × 0.05	0.55 × 0.1 × 0.05

Data collection		
Diffractometer	R-AXIS RAPID	R-AXIS RAPID
No. of measured, independent and observed [*I* > 2σ(*I*)] reflections	12176, 3029, 2035	13902, 3439, 1649
*R*_int_	0.039	0.127

Refinement		
*R*[*F* ^2^ > 2σ(*F* ^2^)], *wR*(*F* ^2^), *S*	0.047, 0.123, 1.02	0.070, 0.180, 0.96
No. of reflections	3029	3439
No. of parameters	195	219
H-atom treatment	H atoms treated by a mixture of independent and constrained refinement	H-atom parameters constrained
Δρ_max_, Δρ_min_ (e Å^−3^)	0.17, −0.20	0.20, −0.24

Computer programs: *PROCESS-AUTO* (Rigaku, 1998 ▸), *SIR2004* (Burla *et al.*, 2005 ▸), *SHELXL2014* (Sheldrick, 2015 ▸), *Mercury* (Macrae *et al.*, 2008 ▸), *WinGX* (Farrugia, 2012 ▸) and *publCIF* (Westrip, 2010 ▸).

## supplementary crystallographic information

### 1-Hydroxy-4-propyloxy-9,10-anthraquinone (I) Crystal data

**Table:**

C_17_H_14_O_4_	*F*(000) = 592
*M**_r_* = 282.28	*D*_x_ = 1.409 Mg m^−^^3^
Monoclinic, *P*2_1_/*n*	Mo *K*α radiation, λ = 0.71073 Å
Hall symbol: -P 2yn	Cell parameters from 8070 reflections
*a* = 4.7354 (3) Å	θ = 3.0–27.4°
*b* = 25.9882 (17) Å	µ = 0.1 mm^−^^1^
*c* = 11.0671 (9) Å	*T* = 200 K
β = 102.268 (7)°	Needle, orange
*V* = 1330.87 (17) Å^3^	0.5 × 0.13 × 0.05 mm
*Z* = 4	

### 1-Hydroxy-4-propyloxy-9,10-anthraquinone (I) Data collection

**Table:**

R-AXIS RAPID diffractometer	2035 reflections with *I* > 2σ(*I*)
Radiation source: normal sealed x-ray tube	*R*_int_ = 0.039
Graphite monochromator	θ_max_ = 27.5°, θ_min_ = 3.0°
Detector resolution: 10 pixels mm^-1^	*h* = −6→5
ω scans	*k* = −33→32
12176 measured reflections	*l* = −14→14
3029 independent reflections	

### 1-Hydroxy-4-propyloxy-9,10-anthraquinone (I) Refinement

**Table:**

Refinement on *F*^2^	Primary atom site location: structure-invariant direct methods
Least-squares matrix: full	Secondary atom site location: structure-invariant direct methods
*R*[*F*^2^ > 2σ(*F*^2^)] = 0.047	Hydrogen site location: mixed
*wR*(*F*^2^) = 0.123	H atoms treated by a mixture of independent and constrained refinement
*S* = 1.02	*w* = 1/[σ^2^(*F*_o_^2^) + (0.0601*P*)^2^ + 0.1583*P*] where *P* = (*F*_o_^2^ + 2*F*_c_^2^)/3
3029 reflections	(Δ/σ)_max_ < 0.001
195 parameters	Δρ_max_ = 0.17 e Å^−^^3^
0 restraints	Δρ_min_ = −0.20 e Å^−^^3^
0 constraints	

### 1-Hydroxy-4-propyloxy-9,10-anthraquinone (I) Special details

**Table:**

Geometry. All esds (except the esd in the dihedral angle between two l.s. planes) are estimated using the full covariance matrix. The cell esds are taken into account individually in the estimation of esds in distances, angles and torsion angles; correlations between esds in cell parameters are only used when they are defined by crystal symmetry. An approximate (isotropic) treatment of cell esds is used for estimating esds involving l.s. planes.

### 1-Hydroxy-4-propyloxy-9,10-anthraquinone (I) Fractional atomic coordinates and isotropic or equivalent isotropic displacement parameters (Å^2^)

**Table:**

	*x*	*y*	*z*	*U*_iso_*/*U*_eq_	
C1	0.5957 (3)	0.24895 (6)	0.42197 (15)	0.0330 (4)	
C2	0.3444 (4)	0.23736 (6)	0.33536 (16)	0.0358 (4)	
H2	0.2979	0.2025	0.314	0.043*	
C3	0.1644 (3)	0.27569 (6)	0.28092 (15)	0.0350 (4)	
H3	−0.0048	0.267	0.2214	0.042*	
C4	0.2236 (3)	0.32769 (6)	0.31091 (15)	0.0304 (3)	
C5	0.4712 (3)	0.34058 (6)	0.40077 (14)	0.0288 (3)	
C6	0.5450 (3)	0.39481 (6)	0.43736 (16)	0.0334 (4)	
C7	0.7983 (3)	0.40429 (6)	0.54037 (15)	0.0319 (4)	
C8	0.8576 (4)	0.45434 (7)	0.58305 (17)	0.0409 (4)	
H8	0.7373	0.4819	0.5465	0.049*	
C9	1.0910 (4)	0.46402 (8)	0.67839 (18)	0.0497 (5)	
H9	1.1314	0.4983	0.7065	0.06*	
C10	1.2665 (4)	0.42437 (8)	0.73352 (19)	0.0512 (5)	
H10	1.4256	0.4314	0.7996	0.061*	
C11	1.2107 (4)	0.37463 (7)	0.69259 (17)	0.0434 (4)	
H11	1.3308	0.3473	0.7306	0.052*	
C12	0.9770 (3)	0.36437 (6)	0.59508 (15)	0.0321 (4)	
C13	0.9209 (3)	0.31127 (6)	0.55027 (15)	0.0315 (4)	
C14	0.6585 (3)	0.30036 (6)	0.45649 (14)	0.0296 (3)	
C15	−0.1815 (3)	0.35323 (7)	0.15327 (15)	0.0375 (4)	
H15A	−0.3207	0.3296	0.1798	0.045*	
H15B	−0.1016	0.336	0.0881	0.045*	
C16	−0.3300 (4)	0.40232 (7)	0.10439 (17)	0.0445 (4)	
H16A	−0.3997	0.4198	0.172	0.053*	
H16B	−0.5009	0.3939	0.0388	0.053*	
C17	−0.1379 (4)	0.43891 (8)	0.0523 (2)	0.0580 (5)	
H17A	−0.0718	0.4222	−0.0161	0.087*	
H17B	0.0296	0.4481	0.1172	0.087*	
H17C	−0.2469	0.4701	0.0221	0.087*	
O1	0.7687 (3)	0.20912 (4)	0.46962 (12)	0.0422 (3)	
O2	0.0480 (2)	0.36605 (4)	0.25698 (11)	0.0374 (3)	
O3	0.4064 (3)	0.43123 (4)	0.38701 (13)	0.0522 (4)	
O4	1.0922 (2)	0.27624 (4)	0.59342 (11)	0.0396 (3)	
H1	0.930 (6)	0.2263 (10)	0.525 (2)	0.086 (8)*	

### 1-Hydroxy-4-propyloxy-9,10-anthraquinone (I) Atomic displacement parameters (Å^2^)

**Table:**

	*U*^11^	*U*^22^	*U*^33^	*U*^12^	*U*^13^	*U*^23^
C1	0.0370 (8)	0.0307 (8)	0.0344 (9)	0.0001 (7)	0.0146 (7)	0.0027 (6)
C2	0.0412 (9)	0.0306 (8)	0.0372 (10)	−0.0049 (7)	0.0122 (7)	−0.0038 (7)
C3	0.0325 (8)	0.0403 (9)	0.0326 (9)	−0.0083 (7)	0.0076 (7)	−0.0063 (7)
C4	0.0279 (7)	0.0336 (8)	0.0299 (9)	−0.0001 (7)	0.0066 (6)	0.0004 (6)
C5	0.0283 (7)	0.0311 (7)	0.0276 (8)	−0.0018 (6)	0.0073 (6)	−0.0001 (6)
C6	0.0332 (8)	0.0307 (8)	0.0352 (9)	0.0002 (7)	0.0049 (7)	−0.0006 (7)
C7	0.0314 (8)	0.0344 (8)	0.0300 (9)	−0.0035 (7)	0.0068 (7)	−0.0012 (7)
C8	0.0418 (9)	0.0356 (8)	0.0436 (11)	−0.0034 (8)	0.0051 (8)	−0.0033 (7)
C9	0.0521 (11)	0.0469 (10)	0.0457 (12)	−0.0109 (9)	0.0003 (9)	−0.0103 (8)
C10	0.0474 (10)	0.0597 (11)	0.0396 (11)	−0.0105 (10)	−0.0059 (8)	−0.0062 (9)
C11	0.0381 (9)	0.0520 (10)	0.0354 (10)	−0.0002 (8)	−0.0026 (7)	0.0033 (8)
C12	0.0305 (8)	0.0371 (8)	0.0283 (9)	−0.0009 (7)	0.0056 (6)	0.0028 (7)
C13	0.0310 (8)	0.0357 (8)	0.0290 (9)	0.0006 (7)	0.0095 (6)	0.0058 (6)
C14	0.0301 (7)	0.0316 (7)	0.0286 (8)	−0.0008 (7)	0.0099 (6)	0.0017 (6)
C15	0.0286 (8)	0.0520 (10)	0.0294 (9)	−0.0023 (8)	0.0003 (7)	−0.0052 (7)
C16	0.0337 (9)	0.0589 (11)	0.0376 (11)	0.0077 (9)	0.0002 (7)	−0.0031 (8)
C17	0.0502 (11)	0.0622 (12)	0.0572 (14)	0.0067 (10)	0.0019 (10)	0.0147 (10)
O1	0.0472 (7)	0.0307 (6)	0.0489 (8)	0.0055 (6)	0.0106 (6)	0.0054 (5)
O2	0.0331 (6)	0.0388 (6)	0.0357 (7)	0.0023 (5)	−0.0028 (5)	−0.0018 (5)
O3	0.0524 (8)	0.0329 (6)	0.0595 (9)	0.0053 (6)	−0.0150 (6)	−0.0012 (6)
O4	0.0382 (6)	0.0392 (6)	0.0394 (7)	0.0067 (5)	0.0038 (5)	0.0076 (5)

### 1-Hydroxy-4-propyloxy-9,10-anthraquinone (I) Geometric parameters (Å, º)

**Table:**

C1—O1	1.3552 (19)	C10—C11	1.376 (3)
C1—C2	1.393 (2)	C10—H10	0.95
C1—C14	1.404 (2)	C11—C12	1.397 (2)
C2—C3	1.365 (2)	C11—H11	0.95
C2—H2	0.95	C12—C13	1.471 (2)
C3—C4	1.406 (2)	C13—O4	1.2449 (18)
C3—H3	0.95	C13—C14	1.468 (2)
C4—O2	1.3531 (18)	C15—O2	1.4422 (19)
C4—C5	1.407 (2)	C15—C16	1.501 (2)
C5—C14	1.424 (2)	C15—H15A	0.99
C5—C6	1.487 (2)	C15—H15B	0.99
C6—O3	1.2172 (19)	C16—C17	1.512 (3)
C6—C7	1.489 (2)	C16—H16A	0.99
C7—C8	1.392 (2)	C16—H16B	0.99
C7—C12	1.393 (2)	C17—H17A	0.98
C8—C9	1.379 (3)	C17—H17B	0.98
C8—H8	0.95	C17—H17C	0.98
C9—C10	1.382 (3)	O1—H1	0.98 (3)
C9—H9	0.95		
			
O1—C1—C2	117.38 (14)	C10—C11—H11	120
O1—C1—C14	123.06 (15)	C12—C11—H11	120
C2—C1—C14	119.56 (15)	C7—C12—C11	120.12 (15)
C3—C2—C1	120.52 (15)	C7—C12—C13	120.16 (14)
C3—C2—H2	119.7	C11—C12—C13	119.72 (15)
C1—C2—H2	119.7	O4—C13—C14	120.98 (14)
C2—C3—C4	121.52 (15)	O4—C13—C12	120.02 (14)
C2—C3—H3	119.2	C14—C13—C12	118.98 (14)
C4—C3—H3	119.2	C1—C14—C5	120.30 (14)
O2—C4—C3	122.08 (14)	C1—C14—C13	118.34 (14)
O2—C4—C5	118.55 (13)	C5—C14—C13	121.36 (13)
C3—C4—C5	119.36 (15)	O2—C15—C16	107.86 (14)
C4—C5—C14	118.68 (13)	O2—C15—H15A	110.1
C4—C5—C6	122.03 (14)	C16—C15—H15A	110.1
C14—C5—C6	119.28 (14)	O2—C15—H15B	110.1
O3—C6—C5	122.65 (15)	C16—C15—H15B	110.1
O3—C6—C7	119.34 (14)	H15A—C15—H15B	108.4
C5—C6—C7	118.00 (14)	C15—C16—C17	113.45 (15)
C8—C7—C12	119.21 (15)	C15—C16—H16A	108.9
C8—C7—C6	119.08 (15)	C17—C16—H16A	108.9
C12—C7—C6	121.71 (14)	C15—C16—H16B	108.9
C9—C8—C7	120.09 (17)	C17—C16—H16B	108.9
C9—C8—H8	120	H16A—C16—H16B	107.7
C7—C8—H8	120	C16—C17—H17A	109.5
C8—C9—C10	120.70 (17)	C16—C17—H17B	109.5
C8—C9—H9	119.6	H17A—C17—H17B	109.5
C10—C9—H9	119.6	C16—C17—H17C	109.5
C11—C10—C9	119.97 (18)	H17A—C17—H17C	109.5
C11—C10—H10	120	H17B—C17—H17C	109.5
C9—C10—H10	120	C1—O1—H1	102.8 (14)
C10—C11—C12	119.90 (17)	C4—O2—C15	118.00 (12)
			
O1—C1—C2—C3	−178.11 (14)	C8—C7—C12—C13	−179.10 (14)
C14—C1—C2—C3	2.2 (2)	C6—C7—C12—C13	0.9 (2)
C1—C2—C3—C4	−0.7 (2)	C10—C11—C12—C7	−0.9 (3)
C2—C3—C4—O2	179.53 (15)	C10—C11—C12—C13	179.01 (16)
C2—C3—C4—C5	−1.2 (2)	C7—C12—C13—O4	175.01 (14)
O2—C4—C5—C14	−179.17 (13)	C11—C12—C13—O4	−4.9 (2)
C3—C4—C5—C14	1.5 (2)	C7—C12—C13—C14	−6.2 (2)
O2—C4—C5—C6	−0.3 (2)	C11—C12—C13—C14	173.89 (15)
C3—C4—C5—C6	−179.55 (14)	O1—C1—C14—C5	178.51 (14)
C4—C5—C6—O3	−4.5 (3)	C2—C1—C14—C5	−1.9 (2)
C14—C5—C6—O3	174.43 (16)	O1—C1—C14—C13	−1.2 (2)
C4—C5—C6—C7	175.19 (14)	C2—C1—C14—C13	178.45 (14)
C14—C5—C6—C7	−5.9 (2)	C4—C5—C14—C1	0.0 (2)
O3—C6—C7—C8	4.9 (2)	C6—C5—C14—C1	−178.97 (14)
C5—C6—C7—C8	−174.80 (14)	C4—C5—C14—C13	179.65 (14)
O3—C6—C7—C12	−175.16 (16)	C6—C5—C14—C13	0.7 (2)
C5—C6—C7—C12	5.2 (2)	O4—C13—C14—C1	3.8 (2)
C12—C7—C8—C9	0.0 (3)	C12—C13—C14—C1	−174.90 (13)
C6—C7—C8—C9	179.96 (16)	O4—C13—C14—C5	−175.84 (14)
C7—C8—C9—C10	−0.7 (3)	C12—C13—C14—C5	5.4 (2)
C8—C9—C10—C11	0.6 (3)	O2—C15—C16—C17	64.4 (2)
C9—C10—C11—C12	0.2 (3)	C3—C4—O2—C15	−9.3 (2)
C8—C7—C12—C11	0.8 (2)	C5—C4—O2—C15	171.45 (14)
C6—C7—C12—C11	−179.20 (15)	C16—C15—O2—C4	−175.86 (13)

### 1-Hydroxy-4-propyloxy-9,10-anthraquinone (I) Hydrogen-bond geometry (Å, º)

**Table:**

*D*—H···*A*	*D*—H	H···*A*	*D*···*A*	*D*—H···*A*
O1—H1···O4	0.98 (3)	1.61 (3)	2.5249 (18)	153 (2)
C8—H8···O3^i^	0.95	2.51	3.270 (2)	137
C15—H15*A*···O1^ii^	0.99	2.88	3.359 (2)	111

Symmetry codes: (i) −*x*+1, −*y*+1, −*z*+1; (ii) *x*−3/2, −*y*+1/2, *z*−1/2.

### 1-Acetyloxy-4-propyloxy-9,10-anthraquinone (II) Crystal data

**Table:**

C_19_H_16_O_5_	*F*(000) = 680
*M**_r_* = 324.32	*D*_x_ = 1.411 Mg m^−^^3^
Monoclinic, *P*2_1_/*c*	Mo *K*α radiation, λ = 0.71073 Å
Hall symbol: -P 2ybc	Cell parameters from 6030 reflections
*a* = 11.7730 (12) Å	θ = 3.2–27.5°
*b* = 15.514 (2) Å	µ = 0.10 mm^−^^1^
*c* = 8.9609 (10) Å	*T* = 200 K
β = 111.153 (8)°	Needle, yellow
*V* = 1526.4 (3) Å^3^	0.55 × 0.1 × 0.05 mm
*Z* = 4	

### 1-Acetyloxy-4-propyloxy-9,10-anthraquinone (II) Data collection

**Table:**

R-AXIS RAPID diffractometer	1649 reflections with *I* > 2σ(*I*)
Radiation source: normal sealed x-ray tube	*R*_int_ = 0.127
Graphite monochromator	θ_max_ = 27.5°, θ_min_ = 3.2°
Detector resolution: 10 pixels mm^-1^	*h* = −15→15
ω scans	*k* = −20→20
13902 measured reflections	*l* = −10→11
3439 independent reflections	

### 1-Acetyloxy-4-propyloxy-9,10-anthraquinone (II) Refinement

**Table:**

Refinement on *F*^2^	Primary atom site location: structure-invariant direct methods
Least-squares matrix: full	Secondary atom site location: structure-invariant direct methods
*R*[*F*^2^ > 2σ(*F*^2^)] = 0.070	Hydrogen site location: inferred from neighbouring sites
*wR*(*F*^2^) = 0.180	H-atom parameters constrained
*S* = 0.96	*w* = 1/[σ^2^(*F*_o_^2^) + (0.0805*P*)^2^] where *P* = (*F*_o_^2^ + 2*F*_c_^2^)/3
3439 reflections	(Δ/σ)_max_ < 0.001
219 parameters	Δρ_max_ = 0.20 e Å^−^^3^
0 restraints	Δρ_min_ = −0.24 e Å^−^^3^
0 constraints	

### 1-Acetyloxy-4-propyloxy-9,10-anthraquinone (II) Special details

**Table:**

Geometry. All esds (except the esd in the dihedral angle between two l.s. planes) are estimated using the full covariance matrix. The cell esds are taken into account individually in the estimation of esds in distances, angles and torsion angles; correlations between esds in cell parameters are only used when they are defined by crystal symmetry. An approximate (isotropic) treatment of cell esds is used for estimating esds involving l.s. planes.

### 1-Acetyloxy-4-propyloxy-9,10-anthraquinone (II) Fractional atomic coordinates and isotropic or equivalent isotropic displacement parameters (Å^2^)

**Table:**

	*x*	*y*	*z*	*U*_iso_*/*U*_eq_	
O1	0.73574 (17)	−0.04963 (13)	0.5929 (3)	0.0468 (6)	
O2	0.42969 (17)	0.16690 (14)	0.7620 (3)	0.0483 (6)	
O3	0.53107 (19)	0.29687 (14)	0.6807 (3)	0.0540 (6)	
O4	0.8033 (2)	0.08785 (16)	0.4753 (3)	0.0702 (8)	
O5	0.9066 (2)	−0.0077 (2)	0.7902 (3)	0.0765 (9)	
C1	0.6653 (2)	0.0082 (2)	0.6418 (4)	0.0408 (7)	
C2	0.5870 (2)	−0.0265 (2)	0.7073 (4)	0.0442 (8)	
H2	0.5871	−0.0869	0.7251	0.053*	
C3	0.5088 (3)	0.0255 (2)	0.7471 (4)	0.0455 (8)	
H3	0.4543	0.0004	0.7912	0.055*	
C4	0.5070 (2)	0.1141 (2)	0.7248 (4)	0.0422 (8)	
C5	0.5900 (2)	0.1519 (2)	0.6615 (3)	0.0372 (7)	
C6	0.5986 (2)	0.2472 (2)	0.6488 (3)	0.0402 (7)	
C7	0.6959 (2)	0.2810 (2)	0.5962 (3)	0.0377 (7)	
C8	0.7145 (3)	0.3696 (2)	0.5977 (4)	0.0444 (8)	
H8	0.6651	0.4071	0.6323	0.053*	
C9	0.8044 (3)	0.4036 (2)	0.5493 (4)	0.0468 (8)	
H9	0.8158	0.4642	0.5501	0.056*	
C10	0.8774 (3)	0.3499 (2)	0.4999 (4)	0.0485 (8)	
H10	0.9396	0.3733	0.468	0.058*	
C11	0.8596 (3)	0.2620 (2)	0.4971 (4)	0.0447 (8)	
H11	0.909	0.225	0.4616	0.054*	
C12	0.7697 (2)	0.2272 (2)	0.5461 (4)	0.0388 (7)	
C13	0.7508 (3)	0.1335 (2)	0.5408 (4)	0.0426 (8)	
C14	0.6682 (2)	0.0963 (2)	0.6175 (3)	0.0379 (7)	
C15	0.8571 (3)	−0.0477 (2)	0.6697 (5)	0.0495 (8)	
C16	0.9202 (3)	−0.1012 (2)	0.5859 (4)	0.0603 (10)	
H16A	0.9495	−0.0642	0.5187	0.09*	
H16B	0.8633	−0.144	0.5187	0.09*	
H16C	0.9894	−0.1308	0.6649	0.09*	
C17	0.3442 (3)	0.1282 (2)	0.8232 (4)	0.0472 (8)	
H17A	0.3879	0.1001	0.9274	0.057*	
H17B	0.2949	0.0841	0.7476	0.057*	
C18	0.2633 (3)	0.1994 (2)	0.8430 (4)	0.0536 (9)	
H18A	0.2249	0.2301	0.7402	0.064*	
H18B	0.3126	0.2413	0.9236	0.064*	
C19	0.1645 (3)	0.1607 (2)	0.8970 (5)	0.0711 (12)	
H19A	0.1176	0.1178	0.8186	0.107*	
H19B	0.1099	0.2066	0.9055	0.107*	
H19C	0.2028	0.1329	1.0015	0.107*	

### 1-Acetyloxy-4-propyloxy-9,10-anthraquinone (II) Atomic displacement parameters (Å^2^)

**Table:**

	*U*^11^	*U*^22^	*U*^33^	*U*^12^	*U*^13^	*U*^23^
O1	0.0506 (11)	0.0288 (13)	0.0650 (15)	0.0007 (9)	0.0258 (12)	−0.0081 (10)
O2	0.0496 (12)	0.0379 (14)	0.0699 (15)	0.0019 (10)	0.0365 (12)	0.0032 (11)
O3	0.0596 (12)	0.0321 (14)	0.0861 (17)	0.0101 (11)	0.0454 (13)	0.0047 (12)
O4	0.1004 (18)	0.0341 (15)	0.109 (2)	0.0120 (13)	0.0781 (18)	0.0053 (14)
O5	0.0604 (14)	0.077 (2)	0.080 (2)	0.0213 (14)	0.0106 (15)	−0.0307 (17)
C1	0.0420 (15)	0.0303 (18)	0.0516 (19)	0.0030 (13)	0.0187 (16)	−0.0029 (15)
C2	0.0465 (15)	0.0286 (19)	0.057 (2)	−0.0033 (13)	0.0179 (17)	0.0010 (14)
C3	0.0460 (16)	0.038 (2)	0.059 (2)	−0.0036 (14)	0.0260 (17)	0.0047 (15)
C4	0.0385 (15)	0.041 (2)	0.0477 (19)	0.0008 (14)	0.0166 (16)	0.0000 (15)
C5	0.0378 (14)	0.0309 (18)	0.0437 (18)	0.0024 (12)	0.0159 (15)	0.0034 (13)
C6	0.0415 (15)	0.0325 (19)	0.049 (2)	0.0023 (14)	0.0195 (16)	0.0016 (14)
C7	0.0410 (15)	0.0293 (18)	0.0436 (18)	0.0054 (13)	0.0163 (15)	0.0024 (13)
C8	0.0529 (17)	0.0319 (19)	0.053 (2)	0.0041 (14)	0.0251 (17)	0.0013 (15)
C9	0.0514 (17)	0.0310 (19)	0.060 (2)	−0.0044 (14)	0.0222 (17)	0.0013 (15)
C10	0.0493 (17)	0.041 (2)	0.061 (2)	−0.0054 (15)	0.0267 (18)	0.0030 (16)
C11	0.0459 (16)	0.037 (2)	0.056 (2)	0.0022 (14)	0.0250 (17)	0.0027 (15)
C12	0.0427 (15)	0.0307 (19)	0.0438 (18)	0.0034 (13)	0.0166 (15)	0.0037 (14)
C13	0.0493 (16)	0.0350 (19)	0.050 (2)	0.0052 (14)	0.0261 (17)	0.0005 (15)
C14	0.0401 (14)	0.0304 (18)	0.0435 (18)	0.0024 (13)	0.0155 (15)	0.0003 (13)
C15	0.0510 (18)	0.032 (2)	0.066 (2)	0.0078 (15)	0.0228 (19)	−0.0013 (17)
C16	0.060 (2)	0.047 (3)	0.080 (3)	0.0102 (17)	0.033 (2)	−0.0139 (19)
C17	0.0456 (16)	0.041 (2)	0.064 (2)	−0.0031 (14)	0.0316 (17)	0.0025 (16)
C18	0.0505 (17)	0.045 (2)	0.077 (2)	0.0002 (15)	0.0368 (19)	−0.0024 (17)
C19	0.071 (2)	0.047 (3)	0.122 (3)	0.0025 (18)	0.067 (3)	−0.004 (2)

### 1-Acetyloxy-4-propyloxy-9,10-anthraquinone (II) Geometric parameters (Å, º)

**Table:**

O1—C15	1.344 (4)	C9—C10	1.380 (4)
O1—C1	1.396 (3)	C9—H9	0.95
O2—C4	1.353 (3)	C10—C11	1.378 (4)
O2—C17	1.440 (3)	C10—H10	0.95
O3—C6	1.213 (3)	C11—C12	1.393 (4)
O4—C13	1.220 (3)	C11—H11	0.95
O5—C15	1.198 (4)	C12—C13	1.469 (4)
C1—C2	1.370 (4)	C13—C14	1.494 (4)
C1—C14	1.387 (4)	C15—C16	1.486 (4)
C2—C3	1.364 (4)	C16—H16A	0.98
C2—H2	0.95	C16—H16B	0.98
C3—C4	1.389 (4)	C16—H16C	0.98
C3—H3	0.95	C17—C18	1.510 (4)
C4—C5	1.421 (4)	C17—H17A	0.99
C5—C14	1.417 (4)	C17—H17B	0.99
C5—C6	1.488 (4)	C18—C19	1.535 (4)
C6—C7	1.484 (4)	C18—H18A	0.99
C7—C8	1.390 (4)	C18—H18B	0.99
C7—C12	1.390 (4)	C19—H19A	0.98
C8—C9	1.385 (4)	C19—H19B	0.98
C8—H8	0.95	C19—H19C	0.98
			
C15—O1—C1	117.9 (2)	C7—C12—C13	120.0 (3)
C4—O2—C17	117.8 (3)	C11—C12—C13	119.8 (3)
C2—C1—C14	121.0 (3)	O4—C13—C12	119.5 (3)
C2—C1—O1	116.7 (3)	O4—C13—C14	121.6 (3)
C14—C1—O1	122.2 (2)	C12—C13—C14	118.9 (2)
C3—C2—C1	120.0 (3)	C1—C14—C5	120.1 (3)
C3—C2—H2	120	C1—C14—C13	120.6 (3)
C1—C2—H2	120	C5—C14—C13	119.3 (3)
C2—C3—C4	121.6 (3)	O5—C15—O1	123.7 (3)
C2—C3—H3	119.2	O5—C15—C16	125.1 (3)
C4—C3—H3	119.2	O1—C15—C16	111.2 (3)
O2—C4—C3	122.7 (3)	C15—C16—H16A	109.5
O2—C4—C5	118.0 (3)	C15—C16—H16B	109.5
C3—C4—C5	119.3 (3)	H16A—C16—H16B	109.5
C14—C5—C4	117.9 (3)	C15—C16—H16C	109.5
C14—C5—C6	121.0 (2)	H16A—C16—H16C	109.5
C4—C5—C6	121.0 (3)	H16B—C16—H16C	109.5
O3—C6—C7	119.8 (3)	O2—C17—C18	107.3 (3)
O3—C6—C5	123.0 (3)	O2—C17—H17A	110.3
C7—C6—C5	117.2 (2)	C18—C17—H17A	110.3
C8—C7—C12	118.8 (3)	O2—C17—H17B	110.3
C8—C7—C6	118.9 (3)	C18—C17—H17B	110.3
C12—C7—C6	122.3 (3)	H17A—C17—H17B	108.5
C9—C8—C7	120.6 (3)	C17—C18—C19	109.5 (3)
C9—C8—H8	119.7	C17—C18—H18A	109.8
C7—C8—H8	119.7	C19—C18—H18A	109.8
C10—C9—C8	120.3 (3)	C17—C18—H18B	109.8
C10—C9—H9	119.8	C19—C18—H18B	109.8
C8—C9—H9	119.8	H18A—C18—H18B	108.2
C11—C10—C9	119.7 (3)	C18—C19—H19A	109.5
C11—C10—H10	120.1	C18—C19—H19B	109.5
C9—C10—H10	120.1	H19A—C19—H19B	109.5
C10—C11—C12	120.3 (3)	C18—C19—H19C	109.5
C10—C11—H11	119.8	H19A—C19—H19C	109.5
C12—C11—H11	119.8	H19B—C19—H19C	109.5
C7—C12—C11	120.2 (3)		
			
C15—O1—C1—C2	−115.1 (3)	C8—C7—C12—C11	−0.6 (4)
C15—O1—C1—C14	68.5 (4)	C6—C7—C12—C11	179.7 (3)
C14—C1—C2—C3	1.2 (5)	C8—C7—C12—C13	−179.0 (3)
O1—C1—C2—C3	−175.2 (3)	C6—C7—C12—C13	1.3 (4)
C1—C2—C3—C4	−0.8 (5)	C10—C11—C12—C7	0.9 (5)
C17—O2—C4—C3	−1.7 (4)	C10—C11—C12—C13	179.3 (3)
C17—O2—C4—C5	178.8 (3)	C7—C12—C13—O4	169.4 (3)
C2—C3—C4—O2	179.4 (3)	C11—C12—C13—O4	−9.0 (5)
C2—C3—C4—C5	−1.1 (5)	C7—C12—C13—C14	−11.2 (4)
O2—C4—C5—C14	−177.9 (3)	C11—C12—C13—C14	170.4 (3)
C3—C4—C5—C14	2.6 (4)	C2—C1—C14—C5	0.3 (5)
O2—C4—C5—C6	4.8 (4)	O1—C1—C14—C5	176.6 (3)
C3—C4—C5—C6	−174.7 (3)	C2—C1—C14—C13	−177.9 (3)
C14—C5—C6—O3	177.8 (3)	O1—C1—C14—C13	−1.7 (5)
C4—C5—C6—O3	−5.1 (5)	C4—C5—C14—C1	−2.2 (4)
C14—C5—C6—C7	−3.0 (4)	C6—C5—C14—C1	175.0 (3)
C4—C5—C6—C7	174.2 (3)	C4—C5—C14—C13	176.1 (3)
O3—C6—C7—C8	5.4 (4)	C6—C5—C14—C13	−6.7 (4)
C5—C6—C7—C8	−173.8 (3)	O4—C13—C14—C1	11.5 (5)
O3—C6—C7—C12	−174.8 (3)	C12—C13—C14—C1	−167.9 (3)
C5—C6—C7—C12	5.9 (4)	O4—C13—C14—C5	−166.7 (3)
C12—C7—C8—C9	0.4 (5)	C12—C13—C14—C5	13.9 (4)
C6—C7—C8—C9	−179.9 (3)	C1—O1—C15—O5	10.3 (5)
C7—C8—C9—C10	−0.5 (5)	C1—O1—C15—C16	−169.8 (3)
C8—C9—C10—C11	0.7 (5)	C4—O2—C17—C18	−175.8 (3)
C9—C10—C11—C12	−0.9 (5)	O2—C17—C18—C19	176.1 (3)

### 1-Acetyloxy-4-propyloxy-9,10-anthraquinone (II) Hydrogen-bond geometry (Å, º)

**Table:**

*D*—H···*A*	*D*—H	H···*A*	*D*···*A*	*D*—H···*A*
C2—H2···O3^i^	0.95	2.6	3.384 (4)	140
C10—H10···O5^ii^	0.95	2.57	3.180 (4)	123

Symmetry codes: (i) −*x*+1, *y*−1/2, −*z*+3/2; (ii) *x*, −*y*+1/2, *z*−1/2.

## References

[bb1] Burla, M. C., Caliandro, R., Camalli, M., Carrozzini, B., Cascarano, G. L., De Caro, L., Giacovazzo, C., Polidori, G. & Spagna, R. (2005). *J. Appl. Cryst.* **38**, 381–388.

[bb2] Farrugia, L. J. (2012). *J. Appl. Cryst.* **45**, 849–854.

[bb3] Furukawa, W., Takehara, M., Inoue, Y. & Kitamura, C. (2014). *Acta Cryst.* E**70**, o1130. 10.1107/S1600536814020996 PMC4257177 25484711

[bb4] Furukawa, W., Takehara, M., Inoue, Y. & Kitamura, C. (2016). *IUCrData*, **1**, x160906.

[bb5] Kitamura, C., Li, S., Takehara, M., Inoue, Y., Ono, K. & Kawase, T. (2015*a*). *Acta Cryst.* E**71**, o504–o505. 10.1107/S2056989015011901 PMC4518951 26279933

[bb6] Kitamura, C., Li, S., Takehara, M., Inoue, Y., Ono, K., Kawase, T. & Fujimoto, K. J. (2015*b*). *Bull. Chem. Soc. Jpn*, **88**, 713–715.

[bb7] Macrae, C. F., Bruno, I. J., Chisholm, J. A., Edgington, P. R., McCabe, P., Pidcock, E., Rodriguez-Monge, L., Taylor, R., van de Streek, J. & Wood, P. A. (2008). *J. Appl. Cryst.* **41**, 466–470.

[bb8] Ohira, N., Takehara, M., Inoue, Y. & Kitamura, C. (2016). *IUCrData*, **1**, x160753.

[bb9] Ohta, A., Hattori, K., Kobayashi, T., Naito, H., Kawase, T. & Kitamura, C. (2012*a*). *Acta Cryst.* E**68**, o2843. 10.1107/S1600536812037361 PMC3470203 23125647

[bb10] Ohta, A., Hattori, K., Kusumoto, Y., Kawase, T., Kobayashi, T., Naito, H. & Kitamura, C. (2012*b*). *Chem. Lett.* **41**, 674–676.

[bb11] Rigaku (1998). *PROCESS-AUTO*. Rigaku Corporation, Tokyo, Japan.

[bb12] Sheldrick, G. M. (2015). *Acta Cryst.* C**71**, 3–8.

[bb13] Westrip, S. P. (2010). *J. Appl. Cryst.* **43**, 920–925.

